# Microhardness of Calcium-enriched Mixture Cement and Covering Glass Ionomers after Different Time Periods of Application

**DOI:** 10.22037/iej.v17i2.37929

**Published:** 2022

**Authors:** Zahra Jaber Ansari, Amir Ghasemi, Hanieh Norozi, Alireza Akbarzade Baghban, Mohammad Samiei

**Affiliations:** a *Department of Operative Dentistry, School of Dentistry, Shahid Beheshti University of Medical Sciences, Tehran, Iran; *; b *Private Practice, Tehran, Iran; *; c *Proteomics Research Center, Department of Biostatistics, School of Allied Medical Sciences, Shahid Beheshti University of Medical Sciences, Tehran, Iran; *; d *Iranian Center for Endodontic Research, Research Institute for Dental Sciences, Shahid Beheshti University of Medical Sciences, Tehran, Iran; *; e *Endodontic Department, Dental School, Tabriz University of Medical Sciences, Tabriz, Iran*

**Keywords:** Calcium-Enriched Mixture, CEM Cement, Glass Ionomer Cement, Tricalcium Silicate, Vital Pulp Therapy, Physical Properties

## Abstract

**Introduction:**

Various studies have recommended using calcium-enriched mixture (CEM) cement in different endodontic treatments, including vital pulp therapy. However, possible reciprocal effects of the covering glass ionomer cement (GIC) on their mechanical properties have not been yet investigated in detail. The current research aimed to experimentally evaluate the surface microhardness of CEM cement and the covering GICs after different application/testing times.

**Materials and Methods:**

Using stainless steel moulds (8×4×4 mm), CEM cement samples were prepared (*n*=120) and randomly divided into 12 experimental groups (*n*=10). CEM cement with thickness of 4 mm was inserted into the moulds, and the remaining spaces were filled with self-cured or light-cured resin-modified GICs at three-time intervals; immediate, in 15 min and after 24 h. Then, the samples were incubated for one and seven days. Using a Vickers microhardness tester, the microhardness of CEM and GICs was measured. The data were analyzed using two-way ANOVA and Tukey’s test, and the significance level was set at 5% (*P*<0.05).

**Results:**

The reciprocal effects of the type/time of application of GICs on the surface microhardness of CEM cement or GICs were statistically significant (*P*<0.001). The surface microhardness of CEM cement and both covering GICs significantly increased over time and in seven-day samples was significantly higher than in one-day samples (*P*<0.05).

**Conclusions:**

Low surface microhardness of CEM/GICs in short-term (24 h) seems transient; and appears to be compensated over a longer period (*i.e*. 7-day). Therefore, using GICs adjacent to CEM cement in single-visit restorative treatments may be advocated.

## Introduction

Vital pulp therapy (VPT) modalities are deliberated as valid treatment options for the management of traumatic injuries and mechanical pulp exposures in primary and permanent teeth [1, 2]; although VPTs have been recently recommended for the treatment of carious pulp exposures [3]. Historically, calcium hydroxide was the biomaterial of choice for pulp capping procedures and pulpotomy; however, approved calcium silicate-based endodontic biomaterials have been lately advocated as first choices for VPTs; *e.g.* mineral trioxide aggregate (MTA), Biodentine and calcium-enriched mixture (CEM) cement [4]. Seemingly, tooth-coloured ProRoot MTA, as the original brand, is able to induce the formation of hydroxyapatite crystals in contact with body tissue fluids and promote hard tissue formation; *i.e.* dentinogenesis [5-7]. Additionally, the optimal desirable properties of ProRoot MTA are not compromised after its clinical application(s); in fact, they are intensified in presence of moisture, making it a highly favourable biomaterial for use in endodontic ministrations. However, it has revealed disadvantages such as high price, difficult manipulation, tooth discolouration, long setting time and solubility [8].

CEM cement is a hydrophilic endodontic biomaterial. It was first introduced in 2006 and could be applied as a substitute for MTA. Calcium oxide is its main component, and the concentrations of other constituents are significantly different from those in the composition of ProRoot MTA. Moreover, its working time is ~5 minutes; with a setting time of 50 min, dimensional change of 0.075 mm, film thickness of 174 μm, flow of 14 mm and pH of 10.71 [9]. The physical properties of this hydrophilic cement seem to improve in presence of moisture. In addition, the biomaterial has shown to promote hydroxyapatite formation via its endogenous calcium and phosphate ions reservoirs [10]. The quality of hydration process during setting reactions of calcium silicate-based biomaterials (*i.e.* MTA and CEM cement) can be evaluated via microhardness testing [11]. 

CEM cement is currently employed in various treatments; comprising, but not limited to, root-end surgery, root/furcal perforation repair, open apices management [apexification, apical plug, revascularisation, apexogenesis (maturogenesis)], root resorption, transplantation/replantation, and different vital pulp therapies (indirect/direct pulp capping, miniature/ partial/full pulpotomy/partial pulpectomy) [12-15]. The benefits of CEM cement consist of bio-sealing ability, biocompatibility, high alkalinity, anti-bacterial/fungal effects and specifically hard tissue induction; *i.e.* dentinogenesis, cementogenesis, and osteogenesis [10, 16-19]. 

The manufacturers of CEM cement recommend that a moist cotton pellet should be placed over the cement after clinical applications, *e.g.* VPTs; and the cavity should be temporarily restored due to the inevitable contact of CEM cement with the covering restorative material. However, this procedure may interfere with the optimal hydration reaction of the biomaterial. Evidence has supported the relation of a tight coronal seal (or hermetic seal) with less microbial microleakage and increased success [20, 21]. In addition, placement of CEM cement and final restoration in one single visit would be highly favourable for both patient and dentist, and would save time/cost. The immediate placement of the final restoration is an important issue for the establishment of coronal seal and assurance of endodontic treatment prognosis [22]. In contrast, the hydrophilic nature of CEM cement has forced clinicians to postpone the final restoration to the next session [19], which increases the cost and frequency of patient visits. 

Glass-ionomer cement (GIC) or resin-modified GIC (RMGI) are commonly used in VPTs as restorative materials. Their application in VPT turns out to be attributed to their adhesion to dental structures/low coefficient of thermal expansion which promotes a good seal [23].

The aim of the present study was to investigate the reciprocal effects of the placement of GIC/RMGI on CEM cement and their mechanical properties after different time periods through the evaluation of surface microhardness of the tested materials.

## Materials and Methods

Sample size was calculated using PASS 11 sample size calculation software; with α=0.05, β=0.1, study power of 90% and previous comparable studies [24, 25]. A total of 120 CEM cement (Bioniqdent, Tehran, Iran) samples were prepared according to the manufacturer’s instructions, inserted into stainless steel moulds (measuring 8×4×4 mm), and randomly divided into 12 following experimental groups (*n*=10):

*Groups 1 and 7:* Conventional GIC (CGIC; LOT 1507025, Equila Forte GC, Alsip, IL, USA) was immediately applied over CEM cement, and the samples were incubated at 37^°^C for 24 h and 7 days. 

*Groups 2 and 8*: CGIC was applied over CEM after 15 min, and the samples were incubated at 37°C for 24 h and 7 days.

*Groups 3 and 9: *CGIC was applied over CEM after 24 h, and the samples were incubated at 37°C for 24 h and 7 days. 

*Groups 4 and 10: *Light-cure RMGI (LOT 1502141; GC Fuji II LC, GC America, Alsip, IL, USA) was immediately applied over CEM, and the samples were incubated at 37°C for 24 h and 7 days.

*Groups 5 and 11: *Light-cure RMGI was applied over CEM cement after 15 min, and the samples were incubated at 37°C for 24 h and 7 days.

*Groups 6 and 12: *Light-cure RMGI was applied over CEM cement after 24 h, and the samples were incubated at 37°C for 24 hours and 7 days.

To test surface microhardness, 50 g load was steadily applied on the samples over 10 sec. Microhardness was measured at 0.5 mm to 1 mm distance from the GIC-CEM cement interface using Vickers hardness tester (Buehler, Lake Bluff, IL, USA). 

The data were analyzed using SPSS version 22. The effects of different times of application of GICs (immediate, after 15 min and after 24 h) on CEM cement, type of GIC (conventional or RMGIC) and duration of incubation (24 h or 7 days) on the surface microhardness of CEM cement/GICs were analysed using two-way ANOVA. In case of significant differences, pairwise comparisons were carried out using Tukey’s test. Level of significance was set at *P*=0.05.

## Results

The mean and standard deviations of surface microhardness of CEM cement and GICs in all experimental groups are presented in Table 1. 

Based on the results of two-way ANOVA in one-day samples, the effects of the type of GICs, time of GICs application, and the interaction effect of “material” and “time of application (ToA)” on the surface microhardness of CEM cement/GICs were statistically significant (*P*<0.001). In seven-day samples, the effects of type of GICs on the surface microhardness of CEM cement or GICs were statistically significant and insignificant, respectively (*P*<0.02 and *P*=0.07). However, the effects of ToA of GICs and the interaction effect of “material” and “ToA” on the surface microhardness of CEM were not statistically significant.

Pairwise comparisons of seven-day samples using Tukey’s test revealed insignificant differences in the surface microhardness of the two tested materials in almost all groups (Table 1). Nonetheless, similar comparisons of one-day samples showed opposite results (inhomogeneous values).

## Discussion

The present *in vitro* study evaluated the surface microhardness of CEM cement covered by GICs as well as the microhardness of GICs after different treatment time periods. The obtained results of the current investigation demonstrated that the mean values of surface microhardness of one-day CEM samples were low and significantly different based on the time of GICs applications. However, the mean values of surface microhardness have significantly/homogeneously increased over 7-day period, with no adverse effect on the ultimate surface microhardness of CEM cement as an important mechanical characteristic of dental biomaterials. Considering that the expeditious effects of GICs on the surface microhardness of CEM cement were well-compensated during the seven-day period, it seems that such short-term deleterious effects could be of minute importance and transient. Therefore, in different clinical applications, *e.g.* VPTs, GICs may be applied over the freshly inserted CEM biomaterial in the same session. Furthermore, similar changes were evident for the surface microhardness of GICs over the tested time; however, the 7-day mean values were insignificantly higher for RMGI in comparison to CGICs.

Setting reaction of GICs has been well studied and depends on the composition of GICs as well as the wetness of environment. The reaction includes the dissolution of peripheral areas of glass silicate particles in the polyacrylic acid-based liquid; resulting in the release of calcium and aluminium ions. Next, calcium ions are chelated with carboxyl groups and produce an amorphous polymer gel. However, calcium ions are slowly replaced with aluminium ions after 24-72 h and thus, a highly cross-linked matrix is created [26].

Water is an important component of GICs and is concurrently produced as a by-product of the GICs setting reaction. It has been shown that the dehydration process decreases the physical properties of conventional GICs [27]. On the contrary, RMGICs are resistant to primary contamination with water since they have an organic matrix and do not need protection during setting [28]. Moreover, the setting reaction of RMGICs is a chain reaction, and therefore, is much faster than that of CGICs. Thus, exposure to moisture seems to have a comparatively minor impact on RMGICs [29]. In the reported study, in all GICs samples, the mean values of surface microhardness increased over time until day 7; which was probably related to the incubation of samples in wet/warm conditions for a long period that led to adequate setting reaction. These findings were in agreement with other researchers on the constant increase in the strength of GICs over time [30].

**Table 1:** The mean and standard deviations of surface microhardness of CEM cement and GICs samples (Kg/mm^2^) in experimental groups

**Type of GICs**	**Time of application of GICs over CEM**	**Time of tests (measurement)**	**Mean (SD)** **(CEM cement)**	**Mean (SD)** **(GICs)**
**CGIC**	Immediately	After one day (24 h)	11.08 (1.94) ^a^	63.49 (4.15) ^a^
	15 min		27.92 (1.61) ^b^	71.08 (6.72) ^b^
	24 h		34.33 (1.94) ^c^	87.23 (7.55) ^c^
**RMGI**	Immediately		23.49 (2.80) ^d^	89.58 (8.07) ^d^
	15 min		28.97 (2.16) ^b^	91.58 (7.25) ^d^
	24 h		37.79 (2.36) e	95.55 (6.84) ^d^
**CGIC**	Immediately	After seven days	43.43 (3.78) ^f^	104.88 (8.19) ^e^
	15 min		46.50 (3.61) ^g^	109.04 (9.58)^ e^
	24 h		48.37 (4.80) ^g^	107.55 (10.17) ^e^
**RMGI**	Immediately		48.88 (5.28) ^g^	111.56 (7.99) ^e^
	15 min		49.72 (5.54) ^g^	111.10 (7.76) ^e^
	24 h		48.34 (4.61) ^g^	111.29 (8.08) ^e^

*GICs, Glass ionomer cements; CGIC, Conventional glass ionomer cement; RMGI, Reinforced modified glass ionomer cement;*

*CEM, Calcium-enriched mixture; SD, Standard deviation; Superscript letters, pairwise t-tests indicated the significance of differences*

On the other hand, CEM cement requires water for its setting reaction. In the current study, all samples were incubated at 37^°^C and 95% humidity prior to the measurement of microhardness. By absorbing water from the humid environment, the setting reaction of CEM cement further progressed and consequently, its microhardness increased. Other researchers have shown that humidity can increase the strength of silicate-based biomaterials, which is in line with our findings [31-34]. It seems that the increase of microhardness of CEM samples is mainly attributed to its setting reaction rather than interactions with covering material, *i.e.* GICs, with similar concepts reported for MTA [35]. 

Vickers hardness test was used for measuring the surface microhardness of GICs [31] and the calcium-silicate based biomaterial [25, 36-40], since this method of measurement is non-destructive and easy to perform. Furthermore, Vickers test indirectly evaluates the setting quality of samples. However, it should be noted that microhardness is one of the many mechanical/physical properties of materials.

It should be noted that the current study had an experimental design. “*In vitro*” studies have limitations, specifically in the simulation of clinical setting; where loads applied in laboratory settings are different from those applied to restorations in a clinical setting. In oral cavity, restorations are subjected to tensile, shear, flexural and complex loads; and thus, the outcomes could be different from the findings in laboratory settings. Moreover, thermal stress, saliva and acids are present in the oral cavity and cannot be perfectly simulated in laboratory settings. Therefore, and considering limitations, generalisation of *in vitro* results to clinical setting must be done with caution. Nonetheless, *in vitro* studies are required for the evaluation/assessment of (bio)materials properties prior to their application in clinical conditions. Additionally, clinical studies are required to confirm the accuracy of the current findings. 

## Conclusions

Considering that the application of GICs, immediately or soon after the placement of CEM cement, has no adverse effect on the long-term surface microhardness of CEM cement/GICs, the treatment may be accomplished within a single session to save time and cost.

## References

[1] Ghoddusi J, Forghani M, Parisay I (2014). New approaches in vital pulp therapy in permanent teeth. Iran Endod J.

[2] Parisay I, Ghoddusi J, Forghani M (2015). A review on vital pulp therapy in primary teeth. Iran Endod J.

[3] AAE Position Statement on Vital Pulp Therapy (2021). J Endod.

[4] Zanini M, Hennequin M, Cousson PY (2019). Which procedures and materials could be applied for full pulpotomy in permanent mature teeth? A systematic review. Acta Odontol Scand.

[5] Asgary S, Parirokh M, Eghbal MJ, Ghoddusi J, Eskandarizadeh A (2006). SEM evaluation of neodentinal bridging after direct pulp protection with mineral trioxide aggregate. Aust Endod J.

[6] Sarkar NK, Caicedo R, Ritwik P, Moiseyeva R, Kawashima I (2005). Physicochemical basis of the biologic properties of mineral trioxide aggregate. J Endod.

[7] Azimi S, Fazlyab M, Sadri D, Saghiri MA, Khosravanifard B, Asgary S (2014). Comparison of pulp response to mineral trioxide aggregate and a bioceramic paste in partial pulpotomy of sound human premolars: a randomized controlled trial. Int Endod J.

[8] Camilleri J (2015). Mineral trioxide aggregate: present and future developments. Endodontic Topics.

[9] Asgary S, Shahabi S, Jafarzadeh T, Amini S, Kheirieh S (2008). The properties of a new endodontic material. Journal of endodontics.

[10] Asgary S, Eghbal MJ, Parirokh M, Ghoddusi J (2009). Effect of two storage solutions on surface topography of two root-end fillings. Aust Endod J.

[11] Yang D-K, Kim S, Park J-W, Kim E, Shin S-J (2018). Different setting conditions affect surface characteristics and microhardness of calcium silicate-based sealers. Scanning.

[12] Asgary S, Ahmadyar M (2013). Vital pulp therapy using calcium-enriched mixture: An evidence-based review. J Conserv Dent.

[13] Nosrat A, Asgary S (2010). Apexogenesis of a symptomatic molar with calcium enriched mixture. Int Endod J.

[14] Asgary S, Fazlyab M, Sabbagh S, Eghbal MJ (2014). Outcomes of different vital pulp therapy techniques on symptomatic permanent teeth: a case series. Iran Endod J.

[15] Asgary S, Eghbal MJ, Bagheban AA (2017). Long-term outcomes of pulpotomy in permanent teeth with irreversible pulpitis: A multi-center randomized controlled trial. Am J Dent.

[16] Nosrat A, Asgary S, Eghbal MJ, Ghoddusi J, Bayat-Movahed S (2011). Calcium-enriched mixture cement as artificial apical barrier: A case series. J Conserv Dent.

[17] Naghavi N, Ghoddusi J, Sadeghnia HR, Asadpour E, Asgary S (2014). Genotoxicity and cytotoxicity of mineral trioxide aggregate and calcium enriched mixture cements on L929 mouse fibroblast cells. Dent Mater J.

[18] Asgary S, Moosavi SH, Yadegari Z, Shahriari S (2012). Cytotoxic effect of MTA and CEM cement in human gingival fibroblast cells Scanning electronic microscope evaluation. N Y State Dent J.

[19] Hasheminia M, Loriaei Nejad S, Asgary S (2010). Sealing Ability of MTA and CEM Cement as Root-End Fillings of Human Teeth in Dry, Saliva or Blood-Contaminated Conditions. Iran Endod J.

[20] Gale MS (2000). Coronal microleakage. Ann R Australas Coll Dent Surg.

[21] Utneja S, Nawal RR, Talwar S, Verma M (2015). Current perspectives of bio-ceramic technology in endodontics: calcium enriched mixture cement-review of its composition, properties and applications. Restor Dent Endod.

[22] Zafar M, Iravani M, Eghbal MJ, Asgary S (2009). Coronal and apical sealing ability of a new endodontic cement. Iran Endod J.

[23] Xie D, Brantley WA, Culbertson BM, Wang G (2000). Mechanical properties and microstructures of glass-ionomer cements. Dent Mater.

[24] Shokouhinejad N, Jafargholizadeh L, Khoshkhounejad M, Nekoofar MH, Raoof M (2014). Surface microhardness of three thicknesses of mineral trioxide aggregate in different setting conditions. Restor Dent Endod.

[25] Tabrizizadeh M, Dabbagh MM, Badrian H, Davoudi A (2015). Microhardness Properties of Mineral Trioxide Aggregate and Calcium-enriched Mixture Cement Plugs at Different Setting Conditions. J Int Oral Health.

[26] Sidhu SK, Nicholson JW (2016). A Review of Glass-Ionomer Cements for Clinical Dentistry. J Funct Biomater.

[27] Mount GJ (Oper Dent. 1994). Buonocore Memorial Lecture. Glass-ionomer cements: past, present and future.

[28] Wilson AD (1990). Resin-modified glass-ionomer cements. Int J Prosthodont.

[29] Wilson AD (1991). Glass-ionomer cement--origins, development and future. Clin Mater.

[30] Crisp S, Lewis BG, Wilson AD (1976). Characterization of glass-ionomer cements Long term hardness and compressive strength. J Dent.

[31] Eid AA, Komabayashi T, Watanabe E, Shiraishi T, Watanabe I (2012). Characterization of the mineral trioxide aggregate-resin modified glass ionomer cement interface in different setting conditions. J Endod.

[32] Gancedo-Caravia L, Garcia-Barbero E (2006). Influence of humidity and setting time on the push-out strength of mineral trioxide aggregate obturations. J Endod.

[33] Nandini S, Ballal S, Kandaswamy D (2007). Influence of glass-ionomer cement on the interface and setting reaction of mineral trioxide aggregate when used as a furcal repair material using laser Raman spectroscopic analysis. J Endod.

[34] Ballal S, Venkateshbabu N, Nandini S, Kandaswamy D (2008). An in vitro study to assess the setting and surface crazing of conventional glass ionomer cement when layered over partially set mineral trioxide aggregate. J Endod.

[35] Camilleri J (2011). Scanning electron microscopic evaluation of the material interface of adjacent layers of dental materials. Dent Mater.

[36] Shahi S, Rahimi S, Yavari HR, Ghasemi N, Rezaie Y, Mirzapour S (2018). Effect of the Bone Graft on the Surface Microhardness of Endodontic Biomaterials. Iran Endod J.

[37] Rahimi S, Shahi S, Torabi Z, Rezaie Y, Ghasemi N, Abolhasani S (2018). The Effect of a Mineralized Bone Graft on the Surface Microhardness of Mineral Trioxide Aggregate and Biodentine. Iran Endod J.

[38] Shojaee NS, Adl A, Jafarpour D, Sobhnamayan F (2018). Effect of Different Water-to-powder Ratios on the Solubility and Microhardness of Calcium-Enriched Mixture Cement. Iran Endod J.

[39] Meraji N, Ahmadi E (2020). The effect of bleaching agents on the microstructure and surface microhardness of three calcium silicate-based barrier materials. Iran Endod J.

[40] Dianat O, Mahdian A, Sanagoo N, Mozayeni MA, Eskandarion S (2019). Setting Time and Surface Microhardness of Mineral Trioxide Aggregate and 1% and 5% Fluoride-Doped Mineral Trioxide Aggregate Mixed with Water and Gel-like Polymer. Iran Endod J.

